# Potassium 2-(*N*-hydroxy­carbamo­yl)acetate monohydrate

**DOI:** 10.1107/S1600536809038434

**Published:** 2009-09-30

**Authors:** Elena V. Prisyazhnaya, Irina Odarich, Igor O. Fritsky, Elżbieta Gumienna-Kontecka, Turganbay S. Iskenderov

**Affiliations:** a Kyiv National University of Construction and Architecture, Department of Chemistry, Povitroflotsky Ave., 31, 03680 Kiev, Ukraine; b National Medical University, Department of General Chemistry, Volodymyrska str. 13, 010004 Kiev, Ukraine; c National Taras Shevchenko University, Department of Chemistry, Volodymyrska str. 64, 01033 Kiev, Ukraine; d Faculty of Chemistry, University of Wrocław, 14 F. Joliot-Curie str., 50-383 Wrocław, Poland; e Karakalpakian University, Department of Chemistry, Universitet Keshesi 1, 742012 Nukus, Uzbekistan

## Abstract

The crystal structure of the title compound, K^+^·C_3_H_4_NO_4_^−^·H_2_O, consists of potassium cations, monoanions of 2-carboxy­acetohydroxamic acid [namely 2-(*N*-hydroxy­carbamo­yl)acetate] and solvent water mol­ecules. The elements of the structure are united in a three-dimensional network by numerous K⋯O coordinate bonds and O—H⋯O and N—H⋯O hydrogen bonds. The coordination sphere of the K^+^ ions may be described as a distorted double capped octa­hedron. Bond lengths and angles are similar to those in related compounds.

## Related literature

For background to hydroxamic acids in biological and coordination chemistry, see: Kaczka *et al.* (1962 ▶); Hershko *et al.* (1992 ▶); Ghio *et al.* (1992 ▶); Shao *et al.* (2004 ▶). For hydroxamic acids as versatile bridging ligands, see: Bodwin *et al.* (2001 ▶); Cutland-Van Noord *et al.* (2002 ▶). For related structures, see: Golenya *et al.* (2007 ▶); Gumienna-Kontecka *et al.* (2007 ▶); Wörl *et al.* (2005 ▶). For K—O bond lengths, see: Świątek-Kozłowska *et al.* (2000 ▶); Mokhir *et al.* (2002 ▶).
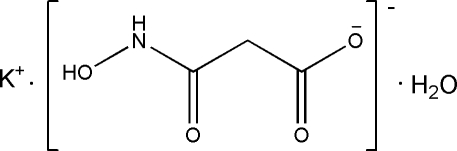


## Experimental

### 

#### Crystal data

K^+^·C_3_H_4_NO_4_^−^·H_2_O
*M* *_r_* = 175.19
Monoclinic, 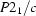

*a* = 7.457 (1) Å
*b* = 13.002 (3) Å
*c* = 6.816 (1) Å
β = 105.41 (3)°
*V* = 637.1 (2) Å^3^
*Z* = 4
Mo *K*α radiation
μ = 0.80 mm^−1^
*T* = 100 K
0.25 × 0.20 × 0.12 mm

#### Data collection

Bruker SMART CCD area-detector diffractometer
Absorption correction: multi-scan (*SADABS*; Sheldrick, 2001 ▶) *T*_min_ = 0.829, *T*_max_ = 0.914
3974 measured reflections
1498 independent reflections
1398 reflections with *I* > 2σ(*I*)
*R*_int_ = 0.025

#### Refinement

*R*[*F* ^2^ > 2σ(*F* ^2^)] = 0.025
*wR*(*F* ^2^) = 0.064
*S* = 1.10
1498 reflections
107 parameters
H atoms treated by a mixture of independent and constrained refinement
Δρ_max_ = 0.36 e Å^−3^
Δρ_min_ = −0.43 e Å^−3^

### 

Data collection: *SMART* (Bruker, 2001 ▶); cell refinement: *SAINT* (Bruker, 1999 ▶); data reduction: *SAINT*; program(s) used to solve structure: *SHELXS97* (Sheldrick, 2008 ▶); program(s) used to refine structure: *SHELXL97* (Sheldrick, 2008 ▶); molecular graphics: *SHELXTL* (Sheldrick, 2008 ▶); software used to prepare material for publication: *SHELXL97*.

## Supplementary Material

Crystal structure: contains datablocks global, I. DOI: 10.1107/S1600536809038434/bv2123sup1.cif

Structure factors: contains datablocks I. DOI: 10.1107/S1600536809038434/bv2123Isup2.hkl

Additional supplementary materials:  crystallographic information; 3D view; checkCIF report

## Figures and Tables

**Table 1:** Hydrogen-bond geometry (Å, °)

*D*—H⋯*A*	*D*—H	H⋯*A*	*D*⋯*A*	*D*—H⋯*A*
O4—H4*O*⋯O1^i^	0.89 (2)	1.79 (2)	2.6820 (13)	177 (2)
N1—H1*N*⋯O2^ii^	0.79 (2)	2.12 (2)	2.9025 (16)	166.7 (18)
O1*W*—H1*W*⋯O2^iii^	0.82 (2)	1.97 (2)	2.7811 (15)	171 (2)
O1*W*—H2*W*⋯O2^iv^	0.84 (3)	1.97 (3)	2.8046 (14)	175 (2)

Symmetry codes: (i) 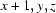; (ii) 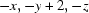; (iii) 
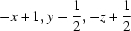
; (iv) 
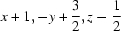
.

**Fig. 1.**
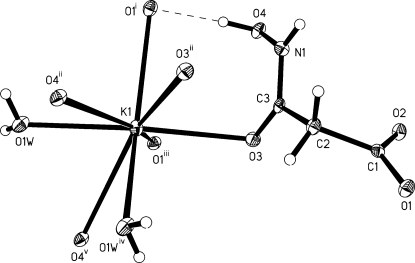
A view of compound (I), with displacement ellipsoids shown at the 50% probability level. H atoms are drawn as spheres of arbitrary radii. The hydrogen bonding is shown by dashed lines [symmetry codes: (i) 1 + x, y, z; (ii) x, 1.5 - y, -1/2 + z; (iii) 1 + x, 1.5 - y, 1/2 + z; (iv)1 - x, 1 - y, - z; (v) 1 - x, -1/2 + y, 1/2 - z].

**Fig. 2.**
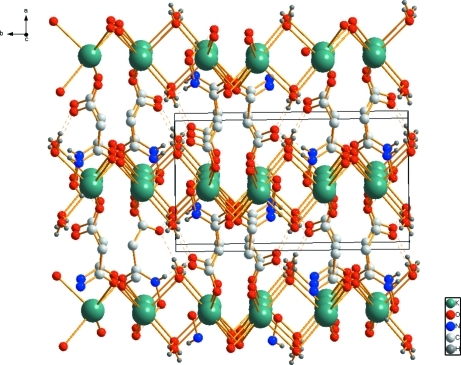
A packing diagram of the title compound. Hydrogen bonds are indicated by dashed lines. H atoms not involved in hydrogen bonding have been omitted for clarity.

## supplementary crystallographic information

### Comment

Hydroxamic acids represent an important class of chelating agents and enzyme inhibitors (Kaczka *et al.*, 1962; Hershko *et al.*, 1992; Ghio *et al.*, 1992; Shao *et al.*, 2004). In recent years hydroxamic acids have also been widely used in coordination chemistry as versatile bridging ligands able to produce multinuclear compounds containing a large number of metal ions, such as metallacrowns (Bodwin *et al.*, 2001; Cutland-Van Noord *et al.*, 2002). Recently we reported that 2-carboxyacetohydroxamic acid is an efficient ligand for obtaining 12-metallacrown-4 complexes with copper(II) ions which can be used as pentanuclear building blocks for preparation of one-dimensional coordination polymers (Gumienna-Kontecka *et al.*, 2007). The present investigation is aimed at the study of the molecular structure of the title compound (I) which is a suitable ligand for preparation of polynuclear complexes and coordination polymers.

The atom-numbering scheme of compound (I) is shown in Fig. 1. The crystal structure of (I) is ionic and consists of potassium cations, monoanions of 2-carboxyacetohydroxamic acid and solvate water molecules. The elements of the structure are united in three-dimensional-network by numerous K···O coordination bonds and the O—H···O and N—H···O hydrogen bonds (Fig. 2,. Table 1).

The residue of 2-carboxyacetohydroxamic acid is a monoanion bearing the deprotonated carboxylic group with the hydroxamic function remaining protonated. The anion exhibits C—O, N—O, C—N bond lengths which are typical for carboxylic and hydroxamic groups (Wörl *et al.*, 2005, Golenya *et al.*, 2007). The conformation of monoanion of 2-carboxyacetohydroxamic acid is significantly non-planar due to the presence of the flexible C—CH_2_—C moiety uniting two planar hydroxamic and carboxylic fragments. The mentioned groups are disposed nearly perpendicularly; the dihedral angle between their planes is equal to 86.37 (5)*^o^*.

The potassium cation exhibits coordination number 8, and its coordination polyhedron can be considered as severely distorted double capped octahedron. Its coordination environment is formed by two solvate water molecules and six oxygen atoms of monoanion of 2-carboxyacetohydroxamic acid belonging to the deprotonated carboxylic groups (O(1)) and both oxygen atoms of the hydroxamic functions(O(3) and O(4)) belonging to the different translational anions. Each potassium cation has in its coordination sphere the oxygen atoms belonging to five different translational monoanions of 2-carboxyacetohydroxamic acid. The K—O bond lengths lie in the range 2.711 (1) - 3.058 (1) Å which is normal for potassium cations (Świątek-Kozłowska *et al.*, 2000; Mokhir *et al.*, 2002).

### Experimental

I was obtained as white powder precipitate by addition of 1 equiv. of KOH (1 *M* aqueous solution) to warm solution of 2-carboxyacetohydroxamic acid (1.19 g, 10 mmol) in water (40 ml) with consequent reduction in volume of the obtained solution. Single crystals suitable for X-ray analysis were grown by slow isothermal evaporation of aqueous solution at room temperature. Anal. For C_3_H_6_NO_5_K (175.18) calcd.: C - 20.57, H - 3.45, N - 8.00. Found: C - 20.7, H - 3.5, N - 7.8. - IR (cm^-1^): 1062 (ν(N—O)), 1380 (ν_s_(COO^-^)), 1580 (ν_as_(COO^-^)), 1672 (ν(C=O) Amide I).

### Refinement

The O—H and N—H H atoms were located from the difference Fourier map and refined isotropically. The methylene H atoms were positioned geometrically and were constrained to ride on their parent atoms, with C—H = 0.975–0.98 Å, and *U*_iso_ = 1.2 *U*_eq_(parent atom).

### Crystal data

**Table:**

K^+^·C_3_H_4_NO_4_^−^·H_2_O	*F*(000) = 360
*M**_r_* = 175.19	*D*_x_ = 1.826 Mg m^−^^3^
Monoclinic, *P*2_1_/*c*	Mo *K*α radiation, λ = 0.71073 Å
Hall symbol: -P 2ybc	Cell parameters from 567 reflections
*a* = 7.457 (1) Å	θ = 3.5–27.5°
*b* = 13.002 (3) Å	µ = 0.80 mm^−^^1^
*c* = 6.816 (1) Å	*T* = 100 K
β = 105.41 (3)°	Needle, colourless
*V* = 637.1 (2) Å^3^	0.25 × 0.20 × 0.12 mm
*Z* = 4	

### Data collection

**Table:**

Bruker SMART CCD area-detector diffractometer	1498 independent reflections
Radiation source: fine-focus sealed tube	1398 reflections with *I* > 2σ(*I*)
graphite	*R*_int_ = 0.025
ω scans	θ_max_ = 28.4°, θ_min_ = 3.5°
Absorption correction: multi-scan (*SADABS*; Sheldrick, 2001)	*h* = −9→9
*T*_min_ = 0.829, *T*_max_ = 0.914	*k* = −17→17
3974 measured reflections	*l* = −6→9

### Refinement

**Table:**

Refinement on *F*^2^	Primary atom site location: structure-invariant direct methods
Least-squares matrix: full	Secondary atom site location: difference Fourier map
*R*[*F*^2^ > 2σ(*F*^2^)] = 0.025	Hydrogen site location: inferred from neighbouring sites
*wR*(*F*^2^) = 0.064	H atoms treated by a mixture of independent and constrained refinement
*S* = 1.10	*w* = 1/[σ^2^(*F*_o_^2^) + (0.0348*P*)^2^ + 0.2753*P*] where *P* = (*F*_o_^2^ + 2*F*_c_^2^)/3
1498 reflections	(Δ/σ)_max_ < 0.001
107 parameters	Δρ_max_ = 0.36 e Å^−^^3^
0 restraints	Δρ_min_ = −0.43 e Å^−^^3^

### Special details

**Table:**

Geometry. All e.s.d.'s (except the e.s.d. in the dihedral angle between two l.s. planes) are estimated using the full covariance matrix. The cell e.s.d.'s are taken into account individually in the estimation of e.s.d.'s in distances, angles and torsion angles; correlations between e.s.d.'s in cell parameters are only used when they are defined by crystal symmetry. An approximate (isotropic) treatment of cell e.s.d.'s is used for estimating e.s.d.'s involving l.s. planes.
Refinement. Refinement of *F*^2^ against ALL reflections. The weighted *R*-factor *wR* and goodness of fit *S* are based on *F*^2^, conventional *R*-factors *R* are based on *F*, with *F* set to zero for negative *F*^2^. The threshold expression of *F*^2^ > σ(*F*^2^) is used only for calculating *R*-factors(gt) *etc*. and is not relevant to the choice of reflections for refinement. *R*-factors based on *F*^2^ are statistically about twice as large as those based on *F*, and *R*- factors based on ALL data will be even larger.

### Fractional atomic coordinates and isotropic or equivalent isotropic displacement parameters (Å^2^)

**Table:**

	*x*	*y*	*z*	*U*_iso_*/*U*_eq_	
K1	0.50362 (4)	0.64050 (2)	0.09788 (4)	0.01167 (10)	
O1	−0.30644 (12)	0.82467 (7)	0.01504 (14)	0.0127 (2)	
O2	−0.09437 (12)	0.92917 (7)	0.20776 (13)	0.01173 (19)	
O3	0.27158 (12)	0.76799 (7)	0.24610 (13)	0.01273 (19)	
O4	0.46616 (12)	0.93401 (7)	0.18090 (13)	0.0122 (2)	
O1W	0.77186 (13)	0.51272 (8)	0.03917 (15)	0.0155 (2)	
N1	0.28881 (15)	0.90970 (9)	0.06042 (16)	0.0110 (2)	
C1	−0.14337 (17)	0.85843 (9)	0.07756 (18)	0.0091 (2)	
C2	0.00201 (17)	0.81466 (10)	−0.02077 (18)	0.0112 (2)	
H2A	−0.0220	0.7419	−0.0464	0.013*	
H2B	−0.0110	0.8482	−0.1510	0.013*	
C3	0.19948 (16)	0.82835 (10)	0.10799 (18)	0.0096 (2)	
H4O	0.543 (3)	0.8999 (18)	0.124 (3)	0.031 (5)*	
H1N	0.243 (3)	0.9497 (15)	−0.027 (3)	0.024 (5)*	
H1W	0.863 (3)	0.4904 (18)	0.125 (3)	0.036 (6)*	
H2W	0.807 (3)	0.5327 (18)	−0.062 (4)	0.043 (6)*	

### Atomic displacement parameters (Å^2^)

**Table:**

	*U*^11^	*U*^22^	*U*^33^	*U*^12^	*U*^13^	*U*^23^
K1	0.01153 (15)	0.01230 (16)	0.01062 (15)	0.00162 (9)	0.00197 (10)	−0.00076 (9)
O1	0.0095 (4)	0.0153 (4)	0.0134 (4)	−0.0009 (3)	0.0029 (3)	−0.0024 (3)
O2	0.0112 (4)	0.0130 (4)	0.0108 (4)	0.0004 (3)	0.0024 (3)	−0.0031 (3)
O3	0.0131 (4)	0.0140 (4)	0.0112 (4)	0.0013 (3)	0.0034 (3)	0.0021 (3)
O4	0.0078 (4)	0.0165 (5)	0.0118 (4)	−0.0021 (3)	0.0020 (3)	−0.0035 (3)
O1W	0.0120 (4)	0.0218 (5)	0.0130 (5)	0.0038 (4)	0.0041 (4)	0.0064 (4)
N1	0.0096 (5)	0.0121 (5)	0.0097 (5)	0.0001 (4)	−0.0002 (4)	0.0006 (4)
C1	0.0103 (5)	0.0096 (6)	0.0070 (5)	0.0016 (4)	0.0018 (4)	0.0026 (4)
C2	0.0104 (5)	0.0133 (6)	0.0099 (5)	−0.0001 (4)	0.0031 (4)	−0.0029 (4)
C3	0.0096 (5)	0.0113 (6)	0.0092 (5)	0.0009 (4)	0.0047 (4)	−0.0028 (4)

### Geometric parameters (Å, °)

**Table:**

K1—O1W	2.7105 (11)	O4—N1	1.3951 (14)
K1—O3	2.7734 (10)	O4—H4O	0.89 (2)
K1—O3^i^	2.8202 (12)	O1W—H1W	0.82 (2)
K1—O1W^ii^	2.8358 (12)	O1W—H2W	0.84 (3)
K1—O1^iii^	2.8558 (12)	N1—C3	1.3351 (17)
K1—O1^iv^	2.9128 (11)	N1—H1N	0.79 (2)
K1—O4^i^	2.9448 (10)	C1—C2	1.5279 (17)
K1—O4^v^	3.0580 (11)	C2—C3	1.5111 (17)
O1—C1	1.2560 (15)	C2—H2A	0.9700
O2—C1	1.2628 (15)	C2—H2B	0.9700
O3—C3	1.2337 (16)		
			
O1W—K1—O3	167.55 (3)	O3^i^—K1—O4^v^	137.54 (3)
O1W—K1—O3^i^	116.31 (3)	O1W^ii^—K1—O4^v^	59.85 (3)
O3—K1—O3^i^	75.90 (2)	O1^iii^—K1—O4^v^	72.32 (3)
O1W—K1—O1W^ii^	91.10 (3)	O1^iv^—K1—O4^v^	147.29 (3)
O3—K1—O1W^ii^	94.15 (3)	O4^i^—K1—O4^v^	99.36 (3)
O3^i^—K1—O1W^ii^	77.83 (4)	N1—O4—H4O	104.5 (13)
O1W—K1—O1^iii^	93.09 (4)	H1W—O1W—H2W	108 (2)
O3—K1—O1^iii^	74.63 (3)	C3—N1—O4	119.52 (10)
O3^i^—K1—O1^iii^	144.05 (3)	C3—N1—H1N	124.0 (14)
O1W^ii^—K1—O1^iii^	124.30 (3)	O4—N1—H1N	116.1 (14)
O1W—K1—O1^iv^	93.40 (3)	O1—C1—O2	124.49 (11)
O3—K1—O1^iv^	87.81 (3)	O1—C1—C2	117.18 (11)
O3^i^—K1—O1^iv^	73.05 (3)	O2—C1—C2	118.23 (11)
O1W^ii^—K1—O1^iv^	149.40 (3)	C3—C2—C1	113.38 (10)
O1^iii^—K1—O1^iv^	85.67 (3)	C3—C2—H2A	108.9
O1W—K1—O4^i^	62.64 (4)	C1—C2—H2A	108.9
O3—K1—O4^i^	129.78 (3)	C3—C2—H2B	108.9
O3^i^—K1—O4^i^	55.86 (3)	C1—C2—H2B	108.9
O1W^ii^—K1—O4^i^	64.93 (3)	H2A—C2—H2B	107.7
O1^iii^—K1—O4^i^	155.21 (3)	O3—C3—N1	123.01 (11)
O1^iv^—K1—O4^i^	90.56 (3)	O3—C3—C2	121.88 (11)
O1W—K1—O4^v^	64.79 (3)	N1—C3—C2	115.11 (11)
O3—K1—O4^v^	108.45 (3)		
			
O1—C1—C2—C3	−159.16 (11)	O4—N1—C3—C2	174.68 (10)
O2—C1—C2—C3	24.31 (15)	C1—C2—C3—O3	83.28 (14)
O4—N1—C3—O3	−5.58 (18)	C1—C2—C3—N1	−96.98 (13)

Symmetry codes: (i) *x*, −*y*+3/2, *z*−1/2; (ii) −*x*+1, −*y*+1, −*z*; (iii) *x*+1, −*y*+3/2, *z*+1/2; (iv) *x*+1, *y*, *z*; (v) −*x*+1, *y*−1/2, −*z*+1/2.

### Hydrogen-bond geometry (Å, °)

**Table:**

*D*—H···*A*	*D*—H	H···*A*	*D*···*A*	*D*—H···*A*
O4—H4O···O1^iv^	0.89 (2)	1.79 (2)	2.6820 (13)	177 (2)
N1—H1N···O2^vi^	0.79 (2)	2.12 (2)	2.9025 (16)	166.7 (18)
O1W—H1W···O2^v^	0.82 (2)	1.97 (2)	2.7811 (15)	171 (2)
O1W—H2W···O2^vii^	0.84 (3)	1.97 (3)	2.8046 (14)	175 (2)

Symmetry codes: (iv) *x*+1, *y*, *z*; (vi) −*x*, −*y*+2, −*z*; (v) −*x*+1, *y*−1/2, −*z*+1/2; (vii) *x*+1, −*y*+3/2, *z*−1/2.
